# Evaluating the significance of platelet count, neutrophil-to-lymphocyte ratio and platelet-to-lymphocyte ratio in critically ill children

**DOI:** 10.1038/s41598-026-63363-9

**Published:** 2026-07-28

**Authors:** Aya Osama Mohamed, Mai Ali Sayed Abdel Ghaney, Yomna Ahmed Hosni

**Affiliations:** 1 Department of Pediatrics, Faculty of Medicine, Cairo University, Cairo, Egypt; 2 Department of Clinical and Chemical Pathology, Faculty of Medicine, Cairo University, Cairo, Egypt

**Keywords:** Neutrophil/Lymphocyte ratio, Platelets/Lymphocyte ratio, Platelets, PICU, Paediatric research, Diseases, Health care

## Abstract

When critically ill pediatric patients are admitted to the pediatric intensive care unit (PICU), they may face significant morbidity and mortality. Therefore, it is essential to identify at-risk patients early to manage their conditions promptly and navigate through the complexities of their illnesses. This study aims to evaluate the prognostic value of platelet count, Neutrophil/Lymphocyte ratio (NLR) and the Platelets/Lymphocyte ratio (PLR) in children undergoing medical care in the PICU.Over a period of 6 months, a prospective study involving 91 PICU patients was conducted. Upon admission, patients underwent detailed history-taking, comprehensive systemic evaluations, and standard laboratory tests that encompassed complete blood counts, C-reactive protein levels, coagulation profiles, as well as liver and kidney function assessments. Subsequently, the NLR and PLR values were determined. The median NLR was slightly elevated in non-survivors compared to survivors, whereas the PLR was higher in survivors, although these differences were not statistically significant. Platelet levels emerged as a predictive factor for mortality, with an AUC of 0.748, a cutoff value of 244.5, and a p-value of 0.002. Additionally, NLR showed a negative correlation with the Glasgow coma score and a positive correlation with age, TLC, CRP, and ALT, with correlation coefficients of (*r*=-0.253,*p* = 0.016), (*r* = 0.320 ,*p* = 0.002), (*r* = 0.292,*p* = 0.005), (*r* = 0.242 ,*p* = 0.027), and (*r* = 0.256 ,*p* = 0.028) respectively. While no statistically significant relationship was observed between NLR and PLR and non-survivors, platelet count showed moderate discriminatory performance for prognostic evaluation and may be used as part of early risk stratification in critically ill patients.

## Introduction

Critical illness can result in organ dysfunction, morbidity, and mortality^1^. System dysfunction, encompassing cardiovascular, renal, neurological, endocrine, and gastrointestinal diseases, is a primary cause for pediatric PICU admissions^2^. Advances in diagnostic and therapeutic methods have improved patient outcomes, underscoring the need for healthcare professionals to identify high-risk patients promptly for effective treatment^3^.

Neutrophils play a pivotal role as the primary circulating leukocytes that safeguard the body against bacterial and fungal infections^4^. Stressful events trigger increases in neutrophil counts, coupled with decreases in lymphocyte counts^5^. The Neutrophil/Lymphocyte ratio (NLR) serves as a marker for subclinical inflammation^6^.

Platelet count serves as an indicator of PICU outcomes and contributes to the calculation of various intensive care unit scores^7,8^. The Platelet/Lymphocyte ratio (PLR) has recently emerged as a valuable inflammatory marker for early intervention^9–11^.

Although NLR and PLR have been employed in adults as predictors of mortality^12–14^, few studies have simultaneously evaluated these biomarkers in pediatrics, particularly in relation to specific disorders. Consequently, this study was conducted to assess the prognostic value of hematological markers including platelet count, NLR and PLR in all critically ill children admitted to the PICU and to validate previous research findings, as prompt and effective management of critically ill patients, which poses a considerable healthcare challenge, remains of utmost importance.

### Patients and methods

This study was a prospective analytical investigation involving critically ill pediatric patients who were admitted to the critical care unit of Children’s Hospital Cairo University over a span of 6 months. The study cohort comprised 91 patients ranging from 1 month to 14 years of age, all presenting various forms of system dysfunction. Patients were excluded if they had primary hematological or immunological conditions, malignancies that could impact CBC parameters, or if there was a history of glucocorticoid use or unavailability of data.

Upon admission, patients underwent a comprehensive evaluation encompassing detailed history-taking (age, gender, reason for admission, and presence of any associated co-morbidities) and a thorough systematic examination, focusing on vital signs (blood pressure, temperature, heart rate, respiratory rate), capillary perfusion, level of consciousness, and identification of any system dysfunction. The severity of illness and mortality risk were assessed using the Pediatric Index of Mortality (PIM 3) score^15^.

Initial laboratory assessments included CBC with differential, CRP, coagulation profile, liver and kidney function tests (LFTs & KFTs), NLR, and PLR. The study documented the outcomes of PICU stay, such as length of stay and mortality.

#### Ethical approval

for the study was obtained from Cairo University’s Ethical Research Committee (N-74–2024), and informed consent was collected from the parent/guardian of each enrolled subject.

### Statistical methods

Sample size was calculated by using Open-epi sample size calculator; with 0.05 alpha error and power of the study 0.95, the anticipated % is 50, the design effect is 1, total number of patients during the study period is 100; accordingly, we will need 80 patients to reject the null hypothesis. We calculated dropout rate at 10%to be 10 patients. So the final sample will be 90 patients.

Coding and data entry were done using The Social Sciences (SPSS) version 28 (IBM Corp., Armonk, NY, USA). Quantitative data was described as mean, standard deviation, median, maximum, and minimum, while categorical data was described using frequency (count) and relative frequency (percentage). The non-parametric Mann-Whitney test was used for the comparison of quantitative variables^16^. Spearman correlation coefficient was utilized for the correlation of quantitative variables^17^. Multivariate logistic regression was performed for mortality prediction using PIM 3 score and platelet count. Receiver operating characteristic (ROC) curve analysis was performed for mortality prediction. P-values below 0.05 were considered statistically significant.

## Results

This prospective study included 91 patients out of a total of 327 patients admitted to our PICU over a 6-month period. The median age of the enrolled cases was 20 months with an IQR of 8–72 months. Table 1 presents the characteristics of surviving and non-surviving patients. Sepsis represented the primary cause for admission, more prevalent in non-surviving patients with a significant p-value of 0.037, followed by chest, neurological, cardiac, renal, and hematological disorders.

**Table 1:** Comparison of characteristics and medical interventions between survivors and non-survivors.

		Non-survivors (*N*=17)		Survivors (*N*=74)		*P* value
Age (in months)	Median	10	(IQR:5–72)	24	(IQR:10–72)	0.235
		**Count**	**%**	**Count**	**%**	
**Gender**	**Male**	9	52.9%	37	50.0%	0.827
	**Female**	8	47.1%	37	50.0%	
**Causes of admission**						
*Sepsis		7	41.2%	11	14.9%	0.037
**Chest**		5	29.4%	35	47.3%	0.180
**Neurological**		4	23.5%	17	23.0%	1
**Cardiac**		3	17.6%	6	8.1%	0.361
**Renal**		1	5.9%	3	4.1%	0.569
**Hematological**		1	5.9%	1	1.4%	0.340
**Other**		0	0.0%	8	10.8%	0.344
**System dysfunction**						
**Neurological**		10	58.8%	20	27.0%	0.012
**Cardiac**		9	52.9%	25	33.8%	0.141
**Chest**		6	35.3%	36	48.6%	0.319
**Renal**		2	11.8%	5	6.8%	0.611
**Gastrointestinal**		2	11.8%	4	5.4%	0.311
**Metabolic**		2	11.8%	6	8.1%	0.640
**Hepatic**		0	0.0%	1	1.4%	1
**Hematological**		1	5.9%	3	4.1%	0.569
**Other**		0	0.0%	1	1.4%	1
Medical interventions in the PICU						
**Ventilation in PICU stay**		12	70.6%	17	23.0%	< 0.001
**Inotropes in PICU stay**		13	76.5%	14	18.9%	< 0.001

Data were presented as count and percentage. PICU: pediatric intensive care unit. IQR; Interquartile Range.

*Sepsis: includes patients with multiple organ system dysfunction and septic shock.

* one patient may have one system or two system affection with no signs of sepsis.

Patients with system dysfunction were primarily of neurological origin, nearly doubling in non-surviving patients 10/17 (58.8%) compared to survivors 20/74 (27%) with a significant p-value of 0.012. Non-survivors required more ventilation and inotropic support, with significant p-values < 0.001 (Table 1).

Table 2 detailed the clinical and laboratory characteristics of the 91 studied patients.

**Table 2:** Comparison of clinical and laboratory characteristics between survivors and non-survivors.

	Non-survivors (*N*=17)		Survivors (*N*=74)				
	Median	IQR	Median		IQR		
**Glascow coma scale (GCS)**	13.00	12.00–15.00	15.00		14.00–15.00		0.002
**PIM 3 score**	6.90	2.00–24.50	1.70		0.50–4.00		0.002
**Hemoglobin (g/dL)**	9.50	8.50–11.20	10.45		8.80–11.90		0.248
**TLC (per µL of blood)**	14.10	7.00–18.60	11.30		7.80–16.10		0.359
**ANC**	9486.00	4752.00–12800.00	7443.00		4332.00–11020.00		0.313
**ALC**	2160.00	1344.00–2766.00	2396.00		1548.00–4060.00		0.336
**NLR**	2.90	1.80–7.80	2.40		1.30–4.50		0.296
**Platelets (per µL of blood)**	153.00	73.00–244.00	335.00		245.00–457.00		0.001
**PLR**	9.12	5.10–21.83	15.10		7.21–22.85		0.163
**CRP (mg/dL)**	28.50	6.00–58.00	12.00		0.00–48.00		0.179
**PC (%)**	61.00	53.00–90.00	89.00		71.00–98.00		0.062
**PT (seconds)**	12.80	12.80–17.00	13.80		12.80–15.00		0.641
**INR**	1.32	1.00–1.70	1.00		1.00–1.20		0.042
**ALT (U/L)**	55.00	27.00–117.00	21.00		13.00–42.00		0.014
**AST (U/L)**	78.00	34.00–197.00	40.50		31.00–65.00		0.221
**Urea (mg/dL)**	34.90	20.00–84.00	23.00		16.00–41.00		0.122
**Creatinine (mg/dL)**	0.60	0.50–0.90	0.40		0.30–0.60		0.002
**Sodium (mmol/L)**	138.00	135.00–145.00	138.00		135.00–141.00		0.637
**Potassium (mmol/L)**	4.50	3.80–5.20	4.30		3.70–5.00		0.337
**PICU length of stay (days)**	9.00	5.00–16.00	8.00		5.00–13.00		0.680

Data were presented as median and IQR.

PIM 3 score: Pediatric index of mortality score, TLC: total leucocytic count, ANC: absolute neutrophilic count, ALC: absolute lymphocytic count, NLR: neutrophil/lymphocyte score, PLR: platelet/lymphocytic score, CRP: C-reactive protein, PC: prothrombin concentration, PT: prothrombin time, PTT: platelet thromboplastin time, INR: international normalized ratio, ALT: alanine aminotransferase, AST: aspartate aminotransferase, PICU: pediatric intensive care unit.

Alk P: alkaline phosphatase, GGT: gamma-glutamyl transferase, pH: potential hydrogen, pCO2: partial pressure of carbon dioxide, HCO3: bicarbonate,.

**Table 3:** Multivariate logistic regression for prediction of mortality using platelets and PIM3.

		P value		OR		95% C.I.			
						Lower		Upper	
**Mortality**	**Platelets**	0.034		0.996		0.992		1.000	
	**PIM 3 score**	0.038		1.049		1.003		1.097	

PIM 3 scores were significantly higher in non-survivors indicating increased baseline severity of illness among these patients.NLR had a median value of 2.90 in non-survivors and 2.40 in survivors, while PLR was higher in survivors, although neither showed statistical significance. However, low platelet count had a significant p-value of 0.001 in non-survivors, in addition to increased INR, ALT, and creatinine levels, with significant p-values of 0.042, 0.014, and 0.002, respectively.

**Fig. 1:**
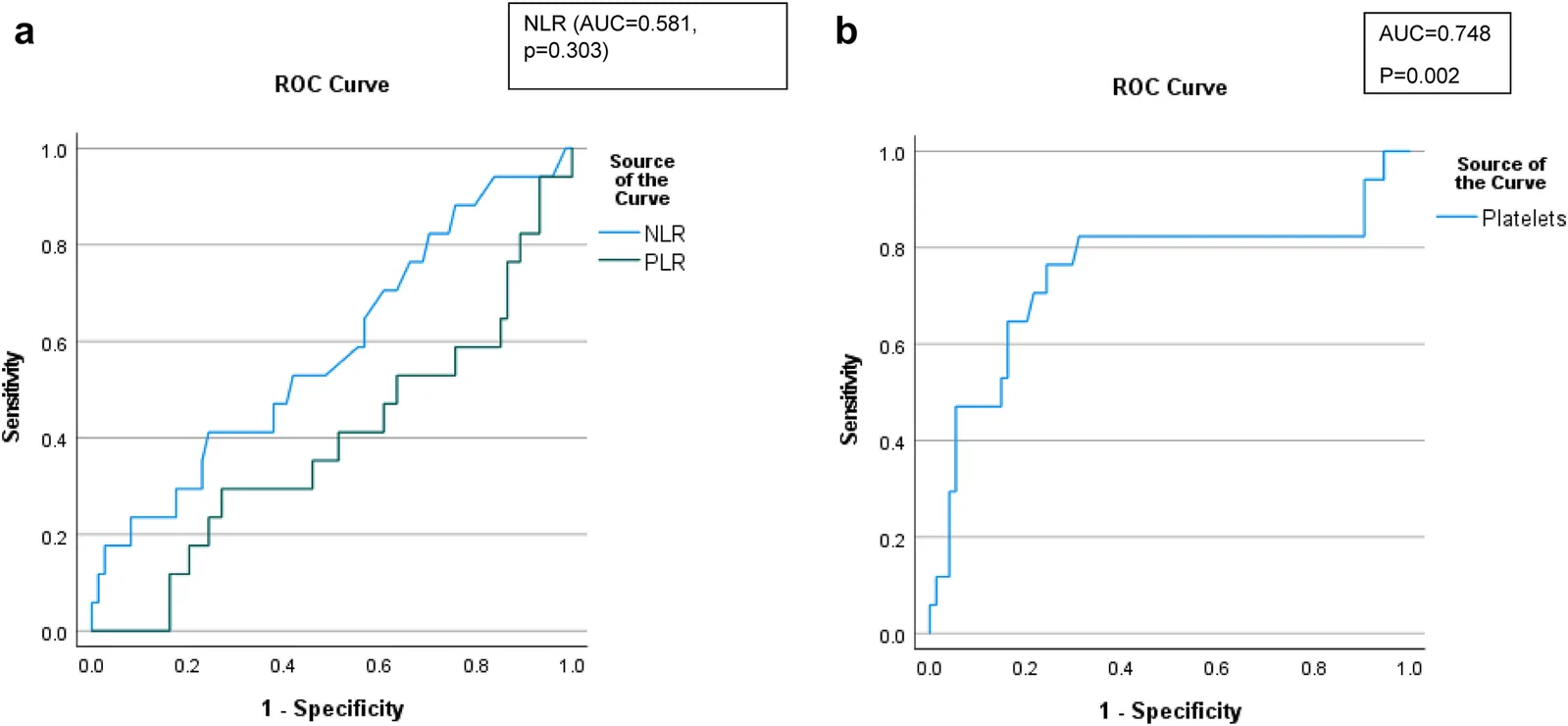
**(a)** ROC curve for NLR and PLR for mortality prediction. **(b)** ROC curve for platelets for prediction of Mortality. **(c)** ROC curve for platelets for prediction of Mortality. ROC: Receiver Operating Characteristic, AUC: Area Under the Curve. r: correlation coefficient.

Moreover, the ROC curve indicated that NLR and PLR were not predictors of mortality, with AUC of 0.561, 0.391, and p-values of 0.453, 0.167, respectively. Conversely, platelets were predictive of mortality, with an AUC of 0.748, a lower bound of 0.589, an upper bound of 0.907, a cutoff value of 244.5, 76.5% sensitivity, 75.7% specificity, and a significant p-value of 0.002 (Fig. 1a and b).Moreover, the PIM3 score had AUC of 0.738, a lower bound of 0.598, an upper bound of 0.879, a cutoff value of 4.3, 64.7% sensitivity, 78.4% specificity, and a significant p-value of 0.003.

**Fig. 2:**
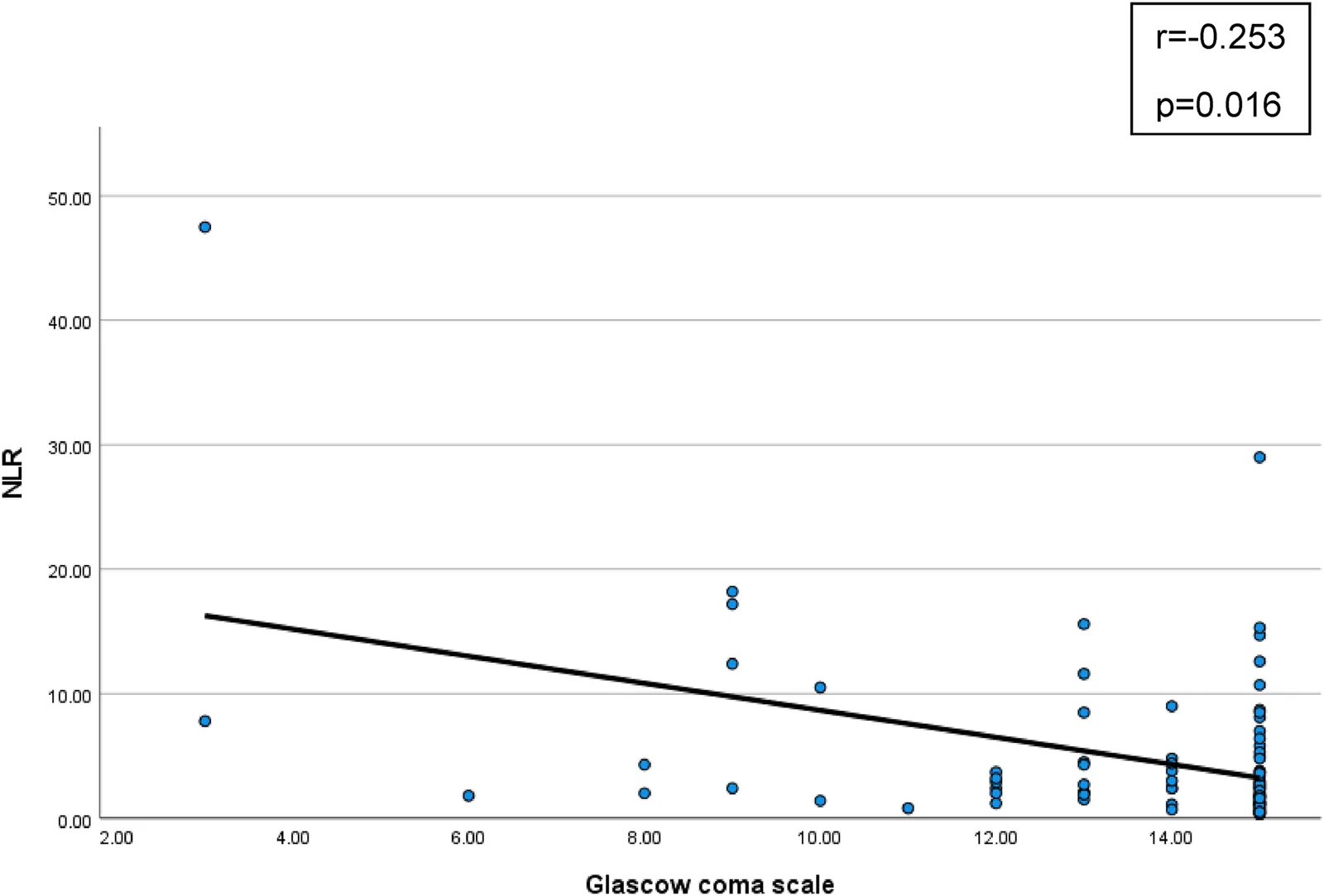
**(a)** ROC curve for NLR and PLR for mortality prediction. **(b)** ROC curve for platelets for prediction of Mortality. **(c)** ROC curve for platelets for prediction of Mortality. ROC: Receiver Operating Characteristic, AUC: Area Under the Curve. r: correlation coefficient.

In the analysis of NLR, PLR, and cohort characteristics, the median NLR was higher (3.45 and IQR 2.00–8.50) in patients with neurological system dysfunction, with a significant p-value of 0.010. Additionally, the median PLR was lower in patients with acute hematological causes (2.31 and IQR 0.20–4.41) and cardiac system dysfunction (median of 8.60 and IQR of 5.55–16.87), with p-values of 0.015 and 0.028, respectively. NLR showed a negative correlation with the Glasgow Coma Score (*r*=−0.253, *p* = 0.016) (Fig. 2). Furthermore, NLR exhibited positive correlations with the patient’s age, TLC, CRP, and ALT with *r* = 0.320, *p* = 0.002; *r* = 0.292, *p* = 0.005; *r* = 0.242, *p* = 0.027; *r* = 0.256, *p* = 0.028, respectively.

## Discussion

Critical illness can result in multiple organ dysfunction^1^. Healthcare providers strive to identify high-risk patients promptly to begin timely management^3^. NLR and PLR were used as mortality predictors in adults^12–14^ with limited data available in pediatrics, prompting our investigation into the prognostic value of hematological markers including platelet count, NLR and PLR in children admitted to the PICU. Our study’s main findings indicated no significant p-value between non-survivors and NLR and PLR, but platelets emerged as a reliable predictor for mortality. Additionally, NLR correlated positively with the age of the patient, GCS, and various laboratory biomarkers such as TLC, CRP, ALT, and creatinine levels.

Among our patients, sepsis was the most common reason for admission in non-surviving patients (41.2%), followed by chest (29.4%) and neurological causes (23.5%). This aligns with a study by Shenoy et al., which highlighted infections as the predominant cause in their study (60.6%) followed by respiratory (12.6%) and neurological causes (10.5%)^18^.

In our study, pulmonary disorders emerged as the second leading cause of mortality in the PICU, following sepsis, in agreement with other studies indicating pneumonia as a significant risk factor for non-survivors and prolonged PICU stays^19–21^.

Examining NLR in non-survivors, our study found a slightly higher median value, consistent with findings from various other studies conducted by Shenoy S et al., Duffy BK et al., Dursun A et al., and Tamelytė E et al., who demonstrated that patients with high NLR had higher rates of mortality, however they demonstrated significant p value^18,22–24^. Also, several studies done in adults as de Jager CP et al. and George AA et al., demonstrated that NLR has a significant association with sepsis and that aligns with our study as the most common cause of non-survivors was suffering from sepsis^25,26^, furthermore a study done by Akilli NB et al. in adults as well detected a strong association between NLR and mortality^27^. Additionally, a study done by Wilar R et al. in neonates recorded that high NLR has a good diagnostic value for early onset sepsis detection^28^. In contrast to our study Yildiz et al., concluded that NLR does not affect 28-day mortality in intensive care patients^29^.

However, our results were not in agreement with other research performed by Saleh et al., and Mısırlıoğlu et al., they found no appreciable difference between survivors and non-survivors and Mısırlıoğlu et al., reported that NLR may not be used as mortality marker^12,30^. This was demonstrated by some adult studies as well^31–33^, where Zheng N et al.^31^ proved that CRP was a better prognostic tool than NLR for mortality detection in patients suffering from pneumonia. While in our study, we could not prove this finding as there was no significant correlation between CRP and survivors and non-survivors.

### In our study

Our study highlighted a positive correlation between NLR and various patient data parameters where NLR correlated positively with the age of the patient, TLC, CRP, ALT, and creatinine levels. Also, a study conducted over 18 months by **Saleh et al.**, in a tertiary center involving all critically ill cases found a positive correlation with TLC but CRP did not show any significance^12^, and a study designed in older population diagnosed with sepsis at an emergency facility showed similar results regarding TLC and CRP^34^.

But up to our knowledge we did not find any studies demonstrating the relation between NLR and ALT and creatinine levels.

Furthermore, non-survivors exhibited higher PIM 3 scores which in accordance with other studies reported that PIM-3 had strong detection for mortality^35,36^, in addition to other scores as Pediatric Risk of Mortality (PRISM) III/IV and Pediatric Logistic Organ Dysfunction-2 (PELOD-2) scores. However, they reported that these scores accuracy may differ significantly across different regions^37,38^. A more recent study done in turkey by Arı HF showed that PRISM III score is suitable for predicting mortality. However, currently there is no gold standard score^39^.

As well non-survivors had a higher Glascow Coma Score with a statistically significant p-value and higher NLR. This is like the study conducted by Shenoy et al., denoting that NLR is higher in critically ill patients, especially those with neurological affection^18^.

On comparing non-survivors versus survivors, they had a lower median of platelets count with a significant p-value of 0.001. Our results are consistent with a prospective study done for 7 months on all critically ill cases admitted to PICU which showed that lower platelet count in non-survivors with a p-value of 0.006^40^.

In addition, our study showed that platelets are predictors of mortality with p-value of 0.002 and we found that no significant difference between PIM3 score and platelets regarding area under curve (Fig. 1c). This aligns with a study done by Pang et al., as they found that reduced platelets is an independent risk factor for PICU mortality^41^.

This concludes a strong association between platelet count and mortality.

In our cohort, the median PLR was higher in survivors. This is in concordance with a study carried out by Pasaribu et al., who concluded that PLR did not significantly change between children with and without sepsis^42^. On the contrary, a retrospective study conducted in Turkey found that non-survivors had a higher PLR and that it is a mortality predictor^30,43^.

In this study, the rate of ventilation was found to be higher among non-survivors at 70.6%, with a statistically significant p-value of less than 0.001. This aligns with findings from a study by Tirkey et al., where the need for ventilation was reported at 54.5%^44^.

Additionally, a cross-sectional study involving 406 patients admitted to the PICU revealed that non-surviving patients had a higher rate of ventilation at 39.34%^45^. A separate study conducted in China with 348 patients showed even higher ventilation rates among non-survivors at 91.3%, also with a significant p-value of less than 0.001^46^.

In terms of inotropic support in the PICU, non-survivors demonstrated a greater requirement for inotropic support, with a rate of 76.5% and a significant p-value of less than 0.001. This trend was consistent with findings from other studies, including the one conducted by Tirkey et al. at 76.4%, and the study by Seifu et al. at 27.43%^44,45^.

It is worth noting the lower median platelet count observed in non-survivors compared to survivors, suggesting platelets as a significant predictor of mortality. Furthermore, non-survivors consistently exhibited higher rates of ventilation and inotropic support, reflecting the severity of their clinical condition compared to survivors.

Despite these insights, it is crucial to consider some limitations of our study. First, it is a small sample size study conducted at a single center. Second, the exclusion criteria were strict to avert patients with comorbidities affecting CBC parameters. Third, a limited number of affordable biomarkers used for comparison and the proposed platelet count cutoff requires external validation before clinical application.

## Conclusion

There was no statistically significant correlation between NLR and PLR and non-survivors, however platelet count showed moderate discriminatory performance for prognostic evaluation. NLR correlated negatively with the Glasgow Coma Scale and positively with the patients’ age, TLC, CRP, and ALT. Additionally, emergency department physicians should keep in mind that no single biomarker could substitute the systemic approach needed in critically ill patients.

## Data Availability

The data sets generated and analyzed during the current study are available from the corresponding author.
